# Cornell University

**Published:** 1897-07

**Authors:** 


### CORNELL UNIVERSITY: GRADUATES IN VETERINARY MEDICINE.

At the commencement at Cornell University, Ithaca, N. Y., June 17, 1897, the following candidates received the degree of

Doctor of Veterinary Medicine: Walter Emerson Howe, V.S., Delhi, N. Y.; Herman Reeve Ryder, V.S., Delhi, N.Y.; Walter Edward Weihe, Wilkesbarre, Pa. The graduation theses of these candidates, based mainly on original study and research, were: "Physiological Temperature of Some Domestic Animals," W. E. Howe; "Physiological Temperature of Some Domestic Animals," H. R. Ryder: "Contagious Pneumonia in the Horse," W. E. Weihe.

The Horace K. White prizes to the most meritorious student in veterinary science and to the second in rank were awarded to Walter Emerson Howe and Herman Reeve Ryder, who were adjudged equal in merit.

